# Implementation of Synthetic Pathways to Foster Microbe-Based Production of Non-Naturally Occurring Carboxylic Acids and Derivatives

**DOI:** 10.3390/jof7121020

**Published:** 2021-11-29

**Authors:** Ana Vila-Santa, Fernão C. Mendes, Frederico C. Ferreira, Kristala L. J. Prather, Nuno P. Mira

**Affiliations:** 1 Institute for Bioengineering and Biosciences, Instituto Superior Técnico, Department of Bioengineering, University of Lisbon, 1049-001 Lisbon, Portugal; ana.vila-santa@ist.utl.pt (A.V.-S.); fernao.mendes@tecnico.ulisboa.pt (F.C.M.); frederico.ferreira@ist.utl.pt (F.C.F.); 2 Associate Laboratory i4HB—Institute for Health and Bioeconomy at Instituto Superior Técnico, Universidade de Lisboa, Av. Rovisco Pais, 1049-001 Lisboa, Portugal; 3 Department of Chemical Engineering, Massachusetts Institute of Technology, Cambridge, MA 02139, USA; kljp@mit.edu

**Keywords:** carboxylic acids, pathway prospecting, microbial cell factories, synthetic biology, levulinic acid, adipic acid, muconic acid, glucaric acid, methacrylic acid

## Abstract

Microbially produced carboxylic acids (CAs) are considered key players in the implementation of more sustainable industrial processes due to their potential to replace a set of oil-derived commodity chemicals. Most CAs are intermediates of microbial central carbon metabolism, and therefore, a biochemical production pathway is described and can be transferred to a host of choice to enable/improve production at an industrial scale. However, for some CAs, the implementation of this approach is difficult, either because they do not occur naturally (as is the case for levulinic acid) or because the described production pathway cannot be easily ported (as it is the case for adipic, muconic or glucaric acids). Synthetic biology has been reshaping the range of molecules that can be produced by microbial cells by setting new-to-nature pathways that leverage on enzyme arrangements not observed in vivo, often in association with the use of substrates that are not enzymes’ natural ones. In this review, we provide an overview of how the establishment of synthetic pathways, assisted by computational tools for metabolic retrobiosynthesis, has been applied to the field of CA production. The translation of these efforts in bridging the gap between the synthesis of CAs and of their more interesting derivatives, often themselves non-naturally occurring molecules, is also reviewed using as case studies the production of methacrylic, methylmethacrylic and poly-lactic acids.

## 1. Overview

**Figure 1:**
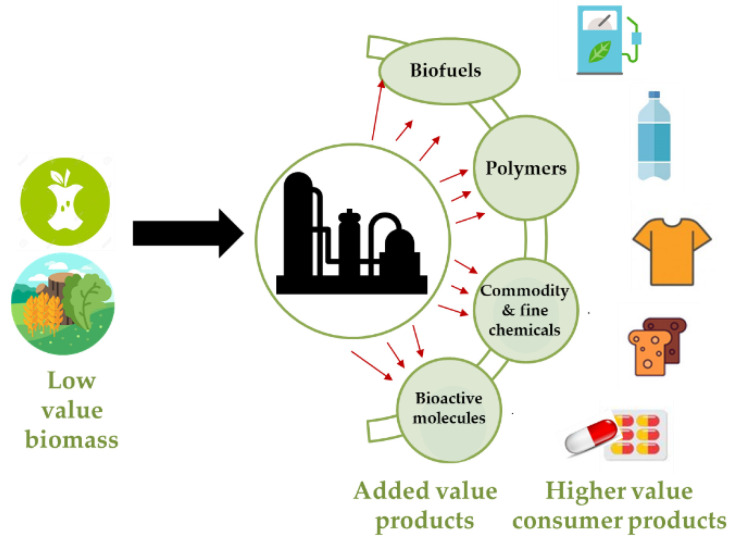
Schematic representation of the diversified set of products that can be produced in a biorefinery, including biofuels, polymers and commodity chemicals.

The development of the consumer goods industry in the last few decades has undoubtedly improved the quality of life in modern societies by providing a range of products that improve our daily routine (such as shampoos, plastic bottles, colorful clothes and amenities like cars or planes). However, it is clear that the way these goods are manufactured is not sustainable, and a dangerous exhaustion of natural resources and irreversible climate changes are envisaged, with unpredictable consequences. Part of the problem concerns the lack of environmental sustainability of the routes used to obtain most of the commodity chemicals necessary for the production of such goods, which is largely dependent on the use of molecules stemming from oil refining. The identification of building block molecules alternative to these oil derivatives that could be obtained by environmentally friendlier routes (for example, by microbial fermentation in a biorefinery) has been suggested as a possible solution [1]. In fact, this idea of having biorefineries producing bulk chemicals (besides biofuels and food/feed products) is gaining interest as it can significantly improve the economic sustainability of these structures [2] (in Figure 1, we provide an overview of this integrated idea of biorefineries providing a diversified set of molecules). The identification of “green” catalysts also provides an alternative to the many industries that nowadays rely on oil derivatives and that may face difficulties in accessing needed precursors for the production of their products in a context that excludes oil refining [2].

Carboxylic acids (CAs) are a cohort of “green” molecules with a recognized potential to displace currently used oil intermediates. In part, this results from their production from biomass being, in most cases, easy to implement, as they are frequent intermediates in central carbon metabolic pathways. Nonetheless, sometimes, the native pathways available for the production of a given CA can be problematic to transfer to a microbial host more amenable for large-scale production. For example, they may require the expression of many heterologous genes (bringing about issues like misfolding or mislocalization of the needed enzymes) or require specific cofactors or intermediates difficult to supply. Incompatibility of selected enzyme properties with the envisaged host(s) physiology (e.g., enzymes that have maximum activity at pHs or temperatures that are non-optimal for the host) is another example of the difficulties that may arise. In other cases that are usually more complex, the CAs fall outside the metabolic repertoire described in biological systems, which means that there is no existing biochemical route to exploit. These issues are not exclusive to CA production. The need to expand the portfolio of molecules produced in microbial cells to include more “non-natural molecules” (such as pharmaceuticals or bulk chemicals other than CAs) is an active field of research in the area of synthetic biology, with various described cases of success [3,4,5,6]. This review focuses on the production of CAs, especially those that are non-natural or whose production using native pathways is challenging (like glucaric, muconic, adipic, levulinic or acrylic acids), emphasizing the efforts made to implement new-to-nature biosynthetic pathways that enable (or significantly improve) their production. Since many of the CA derivatives with industrial relevance are also frequently non-natural, we also review the efforts already made on bridging this bioconversion step. To this end, the production of methacrylic and methylmetacrylic acids, the two main target molecules of itaconic acid, and polylactic acid, one of the main applications envisioned for bio-based produced lactic acid, will be used as representative case studies. In this review, we focused on studies exploring as hosts the bacterium *Escherichia coli* and the yeast *Saccharomyces cerevisiae*. These two microbial species are paradigmatic workhorses in industrial microbiotechnology with a track record of safe use. They are also unique experimental systems having available a panoply of genetic/genomic resources that are unmatched with what is available for other species, an essential asset for synthetic biologists. Additionally, *S. cerevisiae* also has the advantage of allowing the production of CAs at low pH, which allows their recovery in the undissociated form (the one catalytically interesting), thereby significantly reducing the costs associated with downstream processing (as reviewed in [7]).

## 2. Carboxylic Acids and Their Derivatives as “Green Building Blocks”

In 2004, the U.S. Department of Energy (DOE) released a list of the top most attractive “green” monomers that can be obtained from biomass with the potential to be used as building blocks [8]. This list was complemented in 2006, with the results of the European BREW project [8] and again refined in 2010 by the US DOE [9]. CAs are consistently present in the different lists. In Table 1, we compiled those CAs considered more interesting, along with their market applications and derivative targets. All this information fosters research on how to enable or improve the production of CAs in different microbial systems, albeit the research intensity dedicated to each molecule is considerably different (e.g., many studies target production of succinic or lactic acids, while very few focus levulinic acid) (as reviewed by [10]). The potential of CAs as green building blocks comes from their ubiquitous presence across microbial metabolism (for example, they are intermediates of the Krebs cycle or of glycolysis), which renders the easy-to-envision production by fermentation of sugar-enriched biomass. What concerns the exploration of CAs as platform molecules for chemical synthesis stems from them having different functional groups with various, often complementary, properties. For example, dicarboxylic groups (present, for example, in itaconic acid) can be condensed to form polyamides, while keto or hydroxyl groups (present in lactic or gluconic acids) allow the formation of polyesters [11,12]. The global market for CAs was valued at 19.9 billion USD in 2018 with a compound annual growth rate of 8% [13]. The main application segments are food and beverages, animal feed, chemical industry, pharmaceuticals, personal care and agriculture [13]. Among the more interesting CAs, levulinic and 2,5-furandicarboxylic acids are not intermediates in biosynthetic pathways described in biological systems. On the other hand, adipic, acrylic, glucaric and muconic acids have described pathways that, for various reasons, cannot be simply transferred to hosts of interest for microbiotechnology.

## 3. Implementation of New-to-Nature Pathways to Enable Microbe-Based Production of Naturally Occurring CAs

### 3.1. Glucaric Acid

**Figure 2:**
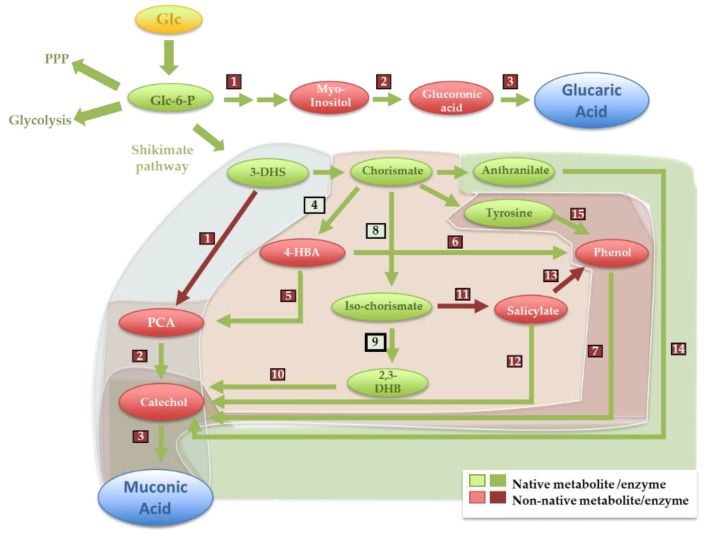
Muconic acid (MA) and glucaric acid (GA) assembled synthetic pathways. Each step is numbered according to the enzyme numbers indicated in Supplementary Table S1. The GA production pathway starts from Glc-6-P and involves the “exogenous” enzymes myo-inositol-1-P synthase (1), myo-inositol oxygenase (2) and uronate dehydrogenase (3). The MA production pathways start from 3-DHS, chorismate, anthranilate or tyrosine. The pathway starting from DHS involves a DHS hydratase (1), a protocatechuate decarboxylase (2) and a catechol 1,2-dioxygenase (3). Five different MA-production pathways originate in chorismate: (i) via 4-HBA, using a chorismate pyruvate lyase (4), a 4-HBA hydrolase (5), a protocatechuate decarboxylase (2) and a catechol 1,2-dioxygenase (3); (ii) via 4-HBA and phenol, using a 4-HBA decarboxylase (6), a phenol hydrolase (7) and a catechol 1,2-dioxygenase (3); (iii) via salicylate, using an isochorismate synthase (8), an isochorismate pyruvate lyase (11), a salicylate monooxygenase (12) and a catechol 1,2-dioxygenase (3); (iv) via salicylate and phenol, using an isochorismate synthase, an isochorismate pyruvate lyase, a salicylate decarboxylase (13) and a phenol hydrolase; (v) via tyrosine, using a tyrosine phenol lyase (15), a phenol hydrolase (7) and a catechol 1,2-dioxygenase (3). The pathway starting from anthranilate (a chorismate derivative) involves an anthranilate 1,2-dioxygenase (14) and a catechol 1,2-dioxygenase. G6P-glucose-6-Phosphate; 3-DHS -3-dehydroshikimate; 4-HBA—4-hydroxybenzoate; 2,3-DHB-2,3-dihydroxybenzoate; Glc-Glucose; Glc-6-P-Glucose-6-phosphate.

Among those CAs that have native pathways described for their production, glucaric and muconic acids represent cases in which the mere transfer of the native pathway into a microbial host is challenging, albeit for different reasons. In the case of glucaric acid, the pathway described is in mammalian cells, and it’s porting to a microbial system would entail the heterologous expression of 10 enzymes [14]. As an alternative, a 5-step synthetic pathway from glucose was assembled in *E. coli*, in which glucose-6-phosphate is converted into glucaric acid using an *S. cerevisiae* myo-inositol synthase, a mammalian myo-inositol oxygenase and a uronate dehydrogenase from *Pseudomonas syringae*, along with two endogenous activities (as detailed in Figure 2) [14].

The implemented synthetic pathway was necessarily inspired by the native production pathway, aiming to undertake the same chemical conversions but recurring to a different portfolio of enzymes. The successful assembly of the glucaric acid-synthetic pathway in *E. coli* overcame two bottlenecks: (i) the production of myo-inositol, which is not produced in this species due to the lack of a myo-inositol synthase; and (ii) the simplification of glucuronic acid to glucaric acid conversion, which in mammalian cells, takes five steps and in the synthetic pathway is mediated in a single step. Improvements to this synthetic pathway were achieved using protein scaffolds (to modulate the effective concentration of myo-inositol at the synthetic complex) [15], fine-tuned regulation of the expression of the myo-inositol synthase [16] or by using different myo-inositol oxygenases [17]. Porting of the initially assembled pathway in *E. coli* to *S. cerevisiae* was proven successful [18,19,20] and recently improved by including a bacterial hemoglobin as a means to improve oxygen availability and, consequently, the activity of myo-inositol oxygenase [21]. In Supplementary Table S1, we provide a comprehensive list of the combinations published in the literature, thus far, of synthetic pathways assembled to produce glucaric acid; we selected to show in Table 2 only those combinations that have resulted in the highest production titers of glucaric acid in *E. coli* or *S. cerevisiae*.

### 3.2. Muconic Acid

Muconic acid is an intermediate in the degradation of aromatic compounds (such as herbicides) prompted by soil bacteria, especially by *Pseudomonas* species [22]. Simple porting of these pathways to more amenable hosts is not trivial since these species would not be equipped with adequate detoxification systems to cope with the deleterious effect of aromatic molecules, often potent xenobiotics. The bacterium *Corynebacterium glutamicum*, a host with established industrial use, was engineered to produce *cis*-muconic acid from ferulic acid, caffeic acid or coumaric acid, three aromatic products produced during the pre-treatment stage of lignin [23,24]. The pathways designed leveraged the use of enzymes described to degrade aromatics, rendering *C. glutamicum* to convert ferulic, caffeic and coumaric acid into a catechol (or a protocatechuate), which is later converted into muconic acid using a catechol dioxygenase [24] (see details in Figure 2). Direct production of muconic acid from fermentable sugars was also implemented in *E. coli* utilizing synthetic pathways, resorting to over-production of dihydroshikimate, chorismate or anthranilate (by engineering of the shikimate pathway), which are then converted to catechol and, ultimately, to muconic acid (as detailed in Figure 2) [24]. Zhang et al. explored the possibility of having the production of the aromatic intermediates and their subsequent degradation assembled in two different *E. coli* strains, thus improving production yield from glucose and enabling the production of muconic acid from various sugar mixtures [25] and from glycerol [26]. The muconic acid production pathways using dihydroshikimic acid have been successfully assembled in vivo in *C. glutamicum* and in *Pseudomonas putida*, resulting in titers around 4-fold higher than those obtained with *E. coli* (as detailed in Supplementary Table S1). The titers obtained using the same pathways assembled in *S. cerevisiae* were, in general, 10-fold lower than the titers obtained in *E. coli*, caused by the limited availability of the prenylated flavin mononucleotide cofactor, essential for the activity of protocatechuate decarboxylase [27]. To overcome this bottleneck, strains with increased activity of phenylacrylic acid decarboxylase or utilizing a protocatechuate decarboxylase from *Talaromyces atroroseus* were engineered (as detailed in Table 2 and in Supplementary Table S1) [28].

## 4. A General View on the Implementation of Synthetic Pathways for the Production of Non-Naturally Occurring Compounds

**Figure 3:**
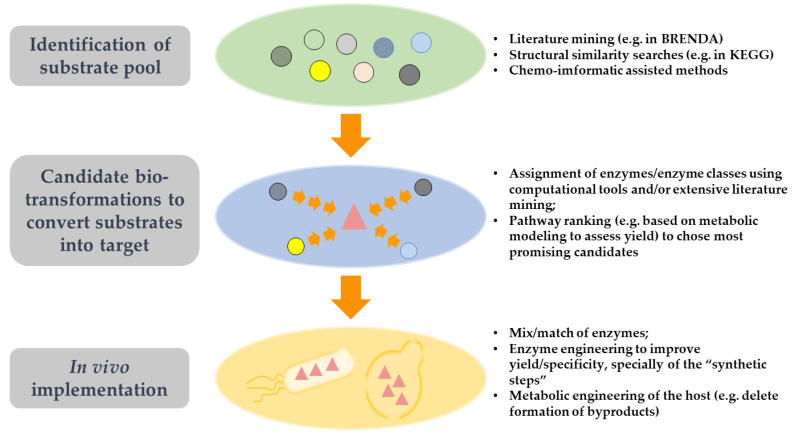
Schematic representation of a putative workflow that can be used to promote the prospection of new-to-nature pathways to enable the production of a non-natural compound in a microbial host of interest (in this case, *S. cerevisiae* and *E. coli*).

When the problem is to settle the production of a “non-natural” compound (in this case, a CA) that is not an intermediate or product of a described pathway, the first challenge is the lack of a route to employ. To solve this, new associations between enzymes and substrates different (but structurally similar) from the native ones are required. Around 37% of the enzymes described in *E. coli* could display promiscuous activity [40], and this greatly increases the number of possible combinations between substrates and enzymes, creating opportunities to “design” reactions that may promote the conversion of a “synthetic” substrate (a substrate different from the native one) into a product of interest (or into a needed intermediate) [41]. The challenge is the identification of the more suitable “synthetic” substrates, the transformations required to transform this “synthetic” substrate into the product of interest, and the identification of available enzyme(s) to mediate those conversions. Figure 3 provides a schematic representation of these “steps” used to prospect synthetic pathways for the production of non-natural molecules, generally settled in a retrobiosynthetic manner since the known goal is the final product of the pathway. This identification of possible associations between synthetic substrates, enzymes and products has been facilitated in the last years by the significant increase in the number of genomic sequences available, accompanied by extensive profiling of endo- and exo-metabolomes. Thus, extensive mining of the literature, for information often dispersed in papers and databases, is the first step to search for reactions that might have the product of interest described as an intermediate or product of a reaction. The search for pathways utilizing metabolites structurally similar to the target is another possible starting point in order to determine a platform biosynthetic pathway to work with. This was, in fact, the approach used to implement the synthesis of the non-natural 2,4-hydroxybutyric acid in *E. coli,* taking advantage of its structural similarity to L-homoserine [42,43]. Despite this, it is not rare that the product of interest has no links with the described pathways, nor does it have useful information published. In these cases, the use of computational tools may be the option to pursue. These tools work in a retrobiosynthetic framework, predicting what can be multiple possible conversions around a desired molecule of interest that is “searched” as a substrate or a product. For that search, the computational tools use a set of reaction rules that are deduced from the extensive study of the rearrangements occurring in atoms and bonds common to the same type of enzymatic reactions [44,45]. Most available computational tools use reaction rules deduced from the analysis of the reactions described in comprehensive databases like the Kyoto Encyclopedia of Genes and Genomes (KEGG) or BRENDA (The Comprehensive Enzyme Information System), giving biological support to what will be the predicted chemical conversions. BNICE (Biochemical Network Integrated Computational Explorer) was the pioneering framework in this field of metabolic retrobiosynthesis [45,46] and laid the groundwork for various tools developed afterwards, like PathPred [47] or UM-BBD [48]. In Supplementary Table S2, we provide a compendium of computational tools available for pathway prospection describing relevant parameters that they work with. Using BNICE reaction rules, the recently released database ATLAS of Biochemistry proposed the repository of the whole theoretical reactome that could be established from the metabolomes described in KEEG [49]. Although this tight connection with the information deposited in databases provides a higher “biological meaningfulness” to the predictions (likely increasing the chance of finding functional associations between enzymes and non-natural substrates), it constrains the prospection of pathways for molecules that are absent from biological portfolios described in KEEG. This is, for example, the case of levulinic or methacrylic acids. To assist this issue, it is possible to resort to computational tools like ReactPred [50], which uses reaction rules deduced from MetaCyc (the Metabolic Pathway Database for all domains of life), or even a set of reaction rules defined/chosen by the user [50]. The downside of using these types of tools, not linked to KEGG, is that they generate a very high number of possible combinations that need to be exhaustively screened by the user to discard unwanted solutions. To reduce the burden, metabolic retrobiosynthesis tools (including those KEGG-dependent) often apply filtering criteria to remove infeasible pathways from the network during the search. For example, Sympheny Biopathway Predictor (a private tool developed in-house by the Genomatica company [4]) does this by limiting the allowed size of the molecule along the biochemical pathway, while GEM-PATH verifies in every iteration (reaction step) of the search if the predicted reactions are thermodynamically feasible and if they have a candidate enzyme to catalyze them [51]. Other filtering criteria include the length of the hypothesized pathway, the number of “synthetic reactions” involved or their thermodynamic feasibility (as detailed in Supplementary Table S2). To complement the identification of the more effective pathways, genome-scale metabolic models have been used to calculate the maximal theoretical yield of the computed pathways and also to anticipate metabolic bottlenecks [52,53].

Besides the identification of the transformations required to convert the pool of substrates into the target molecule, the identification of the most suitable enzymes to perform the envisioned conversion is another critical step that can be facilitated by the use of computational tools. Algorithms utilizing reaction rules deduced from KEGG are able to provide this information because the chemical transformations between substrates and products are, in many cases, associated with a given enzyme class. However, this enzyme assignment is often performed at the third level of the EC classification system, which may contain hundreds of enzymes that have to be manually screened to pinpoint the more interesting candidates. Furthermore, around 20% of all the activities described in the EC classification system remain orphan [54] (that is, they are described but they have no associated protein sequence), resulting in the tools being able to identify the type of enzymes that could be required for the envisaged bioconversions but not being able to pinpoint which enzymes could actually do it. To overcome this limitation, GEM-Path provides to the user the first enzyme homolog in its database that matches each reaction [51], while RetroPath 2.0 considers the number of available enzyme sequences available for the selection of the best-performing candidate pathway with metrics to account for enzyme promiscuity [55].

## 5. Implementation of New-to-Nature Synthetic Pathways to Enable Microbe-Based Production of Adipic, Acrylic and Levulinic Acids

### 5.1. Adipic Acid

**Figure 4:**
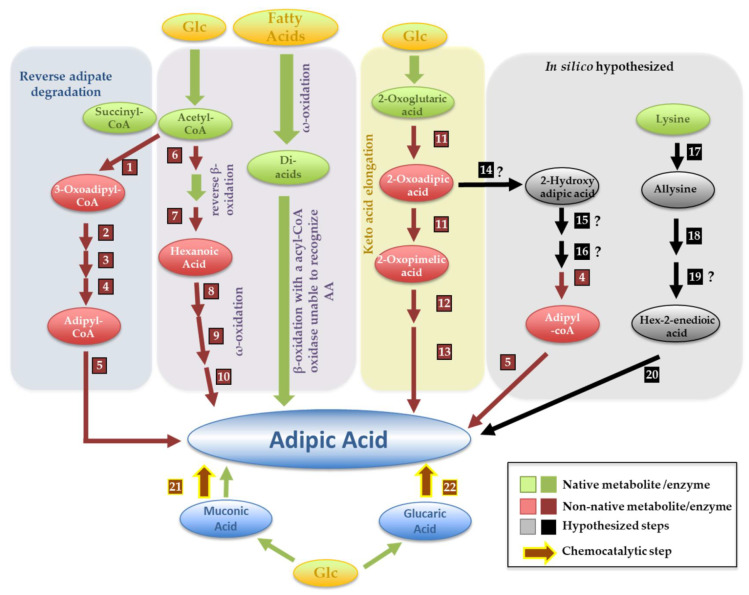
New-to-nature adipic acid production pathways. The pathways depicted in this picture describe the combinations of different enzymes that have been assembled in vivo or suggested in vitro to result in the formation of adipic acid. A detailed description of the enzymes catalyzing the different reactions is provided in Supplementary Table S1. Reactions depicted with “?” have no specific enzyme assigned. Pathways highlighted in yellow (the 2-oxopimelic route), in blue (the reverse adipate route) and in purple (combinations of β, reverse-β and ω-oxidation) have been successfully assembled in vivo, while those shown in the grey (starting from lysine or from 2-oxoadipic acid) result from in silico retrobiosynthesis without experimental validation. The utilization of bio-chemocatalysis in which adipic acid is produced from muconic or glucaric acids obtained by fermentation is also included in the picture. Reverse adipate degradation: (1) 3-oxoadipyl-CoA thiolase; (2) 3-hydroxyadipyl-CoA dehydrogenase; (3) 2,3-dehydroadipyl-CoA hydratase; (4) 2,3-didehydroadipyl-CoA reductase; (5) adipyl-CoA thioesterase; (6) 3-ketoacyl-CoA thiolase, (7) trans-enoyl-CoA reductase; (8) ω-hydroxylase; (9) alcohol dehydrogenase; (10) aldehyde dehydrogenase; (11) entails the multi-step 2-oxoglutaric elongation to 2-oxopimelic acid; (12) branched-chain alpha-ketoacid decarboxylase; (13) endogenous enzyme; (14) 2-hydroxyadipate dehydrogenase, (15) 2-hydroxyadipyl-CoA synthase (15); (16) 2-hydroxyadipyl-CoA dehydratase; (17) lysine conversion to allysine, which is oxidized to 2-aminoadipic acid (18), followed by its conversion to 2-hexenedioic acid (19) and reduction to adipic acid (20). (21) and (22) refer, respectively, to processes of chemical conversion of muconic and glucaric acid to adipic acid.

Adipic acid (or hexanedioic acid) has been essentially regarded as a non-natural compound since the first native pathway describing its production was only in 2015, in the thermophilic bacteria *Thermobifida fusca* [56]. For a long time, the bio-based production of adipic acid relied on the catalytic hydrogenation of muconic or glucaric acids produced by fermentation (as detailed in Figure 4). With the increase in the number of studies reporting the assembly of pathways enabling the production of muconic and glucaric acids in microbes emerged the possibility of performing a full production of adipic acid in vivo. In that sense, in 2018, Raj et al. screened a range of enoyl reductases with described hydrogenation activity over α-unsaturated carboxylic acids to find that the enzyme taken from *Bacillus coagulans* promoted the reduction of muconic acid to adipic acid while also showing high tolerance to oxygen (an identified bottleneck in the activity of these enzymes) and thermostability [35]. Leveraging these results, these authors assembled an entire adipic acid production pathway in *S. cerevisiae* that resulted in a few mg/L of adipic acid [35] (as detailed in Supplementary Table S1). Improvements to this synthetic pathway performed afterwards increased the titers to ~2 mg/L (see details in Supplementary Table S1 and in Table 2). Among the modifications performed in *E. coli*, the use of an enoate reductase from *Clostridium acetobutylicum* was the most successful [57].

Resorting to retrobiosynthetic tools, the companies Verdezyne and Genomatica patented a set of possible adipic acid production pathways [58,59]. The disclosed approaches included the reversal of a β-adipate degradation pathway described in *Penicillium chrysogenum* and the combination of β- and ω-oxidation of fatty acids (as detailed in Figure 4). The reversed adipate degradation pathway got more attention since it had the highest theoretical yield, although this could only be achieved when the precursor succinyl-CoA is derived from the reverse operation of the TCA cycle, a feature that can be challenging to implement in some hosts [53]. Interestingly, the native production pathway described in *T. fusca* ended up being identical to the in silico anticipated reversed adipate degradation pathway [56]. Until now, various combinations of enzymes taken from different hosts have been assembled to reconstruct reversed adipate degradation pathways, with different degrees of success, as detailed in Supplementary Table S1. The best results achieved the g/L scale in *E. coli* using glucose or glycerol as carbon sources (Supplementary Table S1) (as reviewed by [60]). The use of fatty acids degradation as a means to produce adipic acid involves a first ω-oxidation to produce dicarboxylic acids with various lengths. These intermediates undergo β-oxidation and, using an acyl-CoA oxidase, that is unable to recognize adipic acid it is possible to promote the accumulation of this metabolite in the broth (while the other intermediates are channeled for degradation) [59]. A strategy inspired by this combined use of β- and ω-oxidation has also been employed to produce adipic acid from glucose [60]. In this case, acetyl-CoA is elongated to produce hexanoic acid (corresponding to a reversed β-oxidation), which is ω-oxidized to form adipic acid (as detailed in Figure 4). The low specificity of the enzymes used for the ω-oxidation step hampers the channeling of the pathway towards adipic acid production, and most intermediates are instead elongated through the reversed β-oxidation [60].

Two other adipic acid production pathways were raised in the metabolic retrobiosynthesis work described in the patent filled by Genomatica and Verdezyne [58,59], one starting from lysine and the other from 2-oxoadipic acid (see Figure 4). Although both lysine and 2-oxoadipic acid can be easily produced in microbial hosts, these adipic acid-synthesis pathways have not yet been successfully assembled in vivo, likely due to the existence of two steps that do not have an enzyme(s) assigned (as detailed in Figure 4).

### 5.2. Acrylic Acid

Acrylate is a described intermediate in the degradation of the osmolyte dimethylsulfonionpropionate (DMSP) prompted by marine phototrophic bacteria (as reviewed by [61]). Acrylyl-CoA, the CoA ester of acrylate, is also a common metabolic intermediate found, for example, in the fermentation of lactate or alanine by *Clostridium propionicum* or in the 3-hydroxypropionic acid production pathway used for autotrophic CO_2_ fixation. The production of acrylic acid using DMSP as a substrate is infeasible, but acrylyl-CoA has long been identified as a possible anchoring point to establish acrylate-producing pathways (as reviewed by [61]). An initial bottleneck was that the enzymes known to be linked to acrylyl-CoA metabolism were also involved in its catabolism to other intermediates (for example, in lactate catabolism, acrylyl-CoA is rapidly converted to propanoyl-CoA), complicating the acrylyl-CoA to acrylate conversion (as reviewed by [61]). Other problems constraining the production of acrylate from sugars include the low equilibrium constant of the lactate/acrylate equilibrium and the formation of by-products (as reviewed by [61]). Years of research in the field led to the successful in vivo assembly in *E. coli* of three synthetic pathways enabling acrylate production from glycerol, 3-hydroxypropionic acid and β-alanine [36,62,63] (as shown in Table 2 and further detailed in Supplementary Table S1). To successfully enable these synthetic pathways, researchers screened various combinations of possible enzymes leveraging the published information on sequence, affinity and catalytic mechanism of the enzymes described to be involved in the metabolism of acrylyl-CoA or of structurally similar molecules.

### 5.3. Levulinic Acid

**Figure 5:**
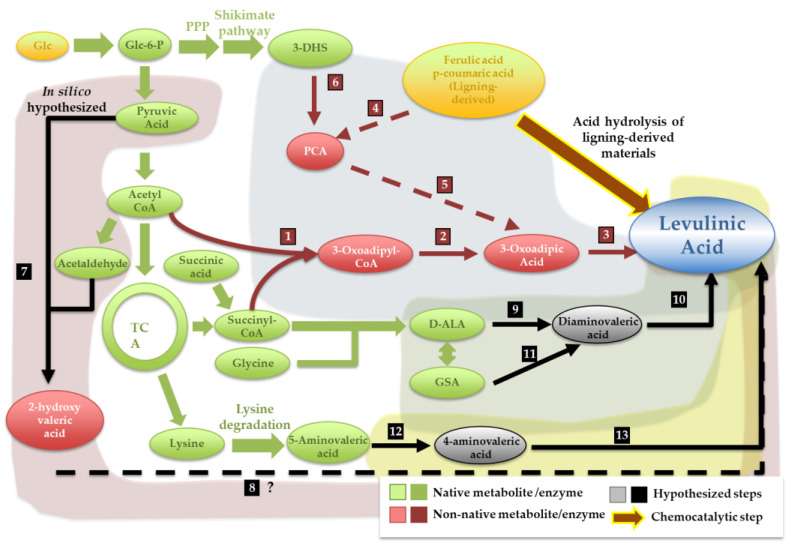
Biosynthetic levulinic acid-production pathways proposed and/or assembled in vivo to enable production in microbial cells. The pathways depicted in the picture include those already assembled in vivo (in blue) and those identified by metabolic retrobiosynthesis (in green, red and yellow). Detailed identification of the enzymes involved in the different steps is provided in Supplementary Table S1. Enzymatic steps are numbered to match the enzymes detailed in Supplementary Table S1. Lumped reactions (involving multiple enzymatic steps) are depicted in dashed lines. For the pathways depicted in blue, one route starts with a β-ketoadipyl-CoA thiolase (1) and a 3-oxoadipyl-CoA transferase (2) and the other route entails the synthesis of protocatechuic acid (PCA) (4) and a dearomatization pathway (5), with both ending with a 3-oxoadipic acid decarboxylase (3). The red pathway entails an aldolase (7) and a series of redox reactions (8), while the green pathway starts with either a D-ALA transaminase (9) or a glutamate semialdehyde aminomutase (11) to yield 4,5-diaminovaleric acid, which is deaminated to LA by diaminovaleric ammonia lyase (10). The yellow pathway includes a 4-amino valeric aminomutase (12) and a 4-aminovaleric transaminase (13).

Unlike adipic and acrylic acids, levulinic acid is not described as an intermediate in any known biochemical pathway. Indeed, levulinic acid was only described as a by-product of the in vitro decarboxylation of α-methylglutamic acid prompted by *E. coli* glutamate decarboxylases [64]. However, α-methylglutamic acid is also a non-natural compound, and its enzymatic conversion to levulinic acid appears to be very difficult, if feasible at all [65]. Recently the production of levulinic acid through fermentation of hemicellulose hydrolysates by a microbial consortium was described; however, neither the metabolic route(s) leading to levulinic acid formation nor the producing species could be identified [66]. The first description of the production of levulinic acid in an engineered microbial host was the work of Cheong et al. in 2016 that explored a platform of Claisen non-decarboxylative condensation reactions to produce various non-natural compounds in *E. coli* [37]. The assembled platform starts with condensation between a primer and an extender unit (e.g., succinyl-CoA and acetyl-coA) to form a CoA activated molecule (e.g., 3-oxoadipyl-coA) that can be subjected to a β-reduction reaction, to enable carbon-chain elongation, or that can undergo a termination reaction to produce a carboxylic acid (like levulinic acid) (as detailed in Figure 5). Despite the success in enabling the production of the different non-natural molecules, in the specific case of levulinic acid, this platform has the problem of requiring a constant supply of succinate in the medium (since succinyl-CoA is used as a substrate in the first reaction), which, together with the low yield, can pose important constraints to the economic viability of the process. Another levulinic acid biosynthetic pathway was also recently assembled in *E. coli* using the degradation of ferulic and p-coumaric acids to produce 3-oxoadipic acid, which is then decarboxylated to yield levulinic acid (as detailed in Figure 5) [38]. Exploring metabolic retrobiosynthesis, a recent study from our group identified five promising levulinic acid production pathways from fermentable sugars (these are the pathways highlighted in grey in Figure 5) [65]. Interestingly, 3-oxoadipic acid was among the identified substrates for levulinic acid production in the computational search performed [38]. Other substrates identified included glutamate semi-aldehyde and δ-aminolevulinic acid, this later substrate being particularly interesting since the underlying pathway entails fewer heterologous steps than the others and is predicted to pose fewer constraints to the endogenous metabolism of yeast or *E. coli* cells [65]. A distinguishable aspect of this in silico study was that it involved not only the use of computational tools based on KEGG reaction rules but also ReactPred and MINEs that go beyond that limitation.

## 6. Bridging the Gap between CAs and Their More Economically Relevant Derivatives through the Assembly of Synthetic Pathways

The market potential of CAs stems from their use as platforms for the production of various bulk chemicals that can afterward be explored by different industries. While the bio-based production of CAs has been receiving a lot of attention, much less has been given on how to enable the production of these derivatives. The more used approach involves the production of the CA in a microbe and its later conversion into the derivative using one (or more) chemical step(s). The above-described production of adipic acid from catalytic hydrogenation of muconic acid and the production of acrylate from microbially produced 3-hydroxypropionic acid [67] or from lactic acid [68] (the strategy pursued by Cargill to implement bio-based production of acrylic acid [69]) are paradigmatic examples of those combined biochemical-chemical approaches. This strategy has the advantage of maintaining flexibility since the same platform molecule is re-routed for multiple applications. However, the conversion steps between the CA and their derivatives can also have a strong environmental impact (for example, by using or producing hazardous reagents or residues), and it may also face problems concerning the purity of the product (for example, if it results in the formation of racemic mixtures that can be hard to separate) that complicates downstream processing. For those CAs in which economic turnover is narrow, these additional problems can pose serious constraints to industrial implementation. In this sense, the possibility of producing of these derivatives directly in those cells already used to produce the precursor CA can be a strategy worthy of attention. The following sections detail a few non-natural CA derivatives whose microbial production has been enabled or for which there are proposals to be enabled exploring synthetic pathways. The case studies presented were selected because the underlying molecules represent very large portions of the projected addressable markets of the precursor CAs (and therefore, for these CAs, there is less margin to leverage the multiple platform approach).

### 6.1. Poly-Lactate Polymers

**Figure 6:**
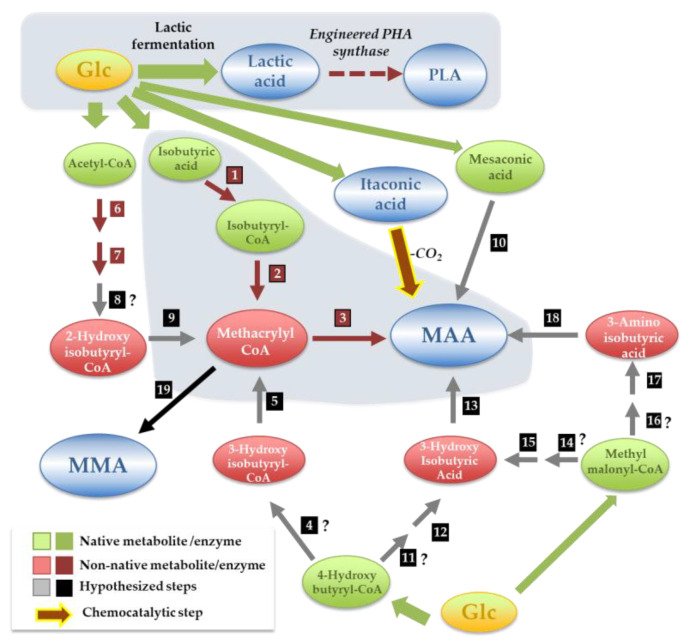
Synthetic poly-lactic acid (PLA), methacrylic acid (MAA) and methyl-methacrylic acid (MMA) production pathways assembled in vivo or identified by in silico metabolic retrobiosynthesis. Detailed information about the enzymes involved in the different steps is provided in Supplementary Table S1. Steps identified by metabolic retrobiosynthesis are indicated in black boxes, and those in which it was not possible to assign an enzyme are marked with “?”. Bio-chemical catalytic conversion of itaconic acid (ITA) to MAA is also shown in the picture as an alternative production method. (1) isobutyryl-CoA synthase; (2) acyl-CoA oxidase; (3) hydroxybenzoyl-CoA thioesterase; (4) 4-hydroxyisobutyryl-CoA mutase; (5) 3-hydroxyisobutyryl-CoA dehydratase; (6) acetoacetyl-CoA thiolase; (7) acetoacetyl-CoA; (8) 3-hydroxyisobutyrate mutase; (9) 2-hydroxyisobutyryl-CoA de-hydratase; (10) mesaconate decarboxylase; (11) 4-hydroxyisobutyryl-CoA mutase; (12) 3-hydroxyisobutyryl-CoA hydrolase; (13) 3-hydroxyisobutyrate dehydratase; (14) methylmalo-nyl-CoA reductase; (15) hydroxyisobutyrate dehydrogenase; (16) methylmalonyl-CoA Reductase; (17) 3-aminoisobutyrate transaminase; (18) 3-aminoisobutyrate ammonia lyase.

Poly-lactate polymers (PLA) occupy a large segment of the applications envisaged for lactic acid, with a particularly interesting rise in its use in the biomedical field due to their biocompatibility (as reviewed by [70,71]). Chemical PLA synthesis is complex, as it involves multiple steps and utilizes hazardous reagents [70,71]. Inspired by the structural similarity between PLA and polyhydroxyalkanoates (PHAs), the microbe-driven synthesis of PLA was envisaged (as detailed in Figure 6). This synthetic pathway leveraged the simplicity of the PHA-production pathway that encompasses only the use of a synthase to polymerize hydroxyacyl-CoAs units. Lactate was the obvious monomer to use; however, the lack of PHA synthases capable of promoting the activation of lactate to lactoyl-CoA posed a serious constraint. Utilizing enzyme evolution approaches, Yang et al. (2010) were able to overcome this problem and assembled a pathway for the production of PLA in *E. coli*, either as a homopolymer or as a heteropolymer with 3-hydroxybutyrate (3HB-co-LA) (Figure 6) [72,73]. Various studies published after leveraged this approach to enable the production of PLAs and their copolymers in other bacterial species and yeasts [74,75].

### 6.2. Methacrylic and Methyl-Methacrylic Acids

Methacrylic acid (MAA) and methyl-methacrylic acid (MMA) represent around 70% of the addressable market projected for itaconic acid [76,77]. The strongest potential of MAA and MMA as platform molecules relies on their high reactivity caused by the polarization of the double bond that enables nucleophilic additions, esterifications, transesterifications and, most importantly, polymerization reactions (catalyzed by heating or by free radical initiators) [78]. MMA is used to produce poly-MMA, which is a transparent and UV-resistant polymer with the commercial name of Plexiglas^®^ that is used in the assembly of electronics, car components, lights, signs and displays. Besides the homopolymer, MMA is also used as a co-monomer in various blends of polymers for use in construction, paints, coatings, automotive components and biomedical materials [79]. A particularly interesting co-polymerization involves 2-hydroxyethyl-metyl acrylic acid (a derivative of MAA) and itaconic acid, resulting in the formation of hydrogels with high biocompatibility and enhanced responsiveness to pH and temperature, interesting to use in drug delivery or as a “biological glue” in wound dressings [80,81]. Today MMA industrial production is undertaken via the acetone cyanohydrin (ACH) route, which has the disadvantage of producing large quantities of ammonium bisulfate, a toxic and costly method to deal with residue [82]. The need for a large supply of hydrogen cyanide, highly hazardous, is another major bottleneck of the ACH route [82]. An alternative has been developed by the Lucite company, in which MAA is produced from syngas and ethylene [83,84]. However, although syngas can be obtained from renewable resources, this “greener” ACH pathway still requires the manipulation of formalin and methanol and the usage of high volumes of low-cost ethylene, which is obtained from steam cracking of fossil fuel [83,84]. These issues have been pushing the study of the bio-based production of MAA and MMA, eventually using itaconic acid as a precursor. Indeed, itaconic acid can be chemically decarboxylated to MAA; however, this requires extreme temperatures and pressures [39]. Production of MAA by chemical decarboxylation of citric acid, citramalic acid or 2-hydroxyisobutyric acid, all these being CAs with implemented production routes in microbes [85,86], has also been suggested as possible alternatives, but these can require several steps and the use of supercritical or near-critical water systems [87] (as reviewed by [88]).

MAA and MMA are not produced by biological systems, but methacrylyl-CoA is an intermediate in the valine degradation pathway used by some bacterial and fungal species. Leveraging this and using metabolic retrobiosynthesis, Genomatica has filed in a patent describing a set of possible production pathways for MAA or MMA starting from 3-aminoisobutyrate, 3-hydroxyisobutyrate, mesaconate or methacrylyl-CoA (as detailed in Figure 6) [89]. For several of the synthetic steps, it was not possible to assign an enzyme, complicating the subsequent assembly of the pathway in vivo. More recently, Lucite has also filed a patent describing the assembly of a synthetic pathway to produce MAA from isobutyric acid using an acyl-CoA oxidase from *Arabidopsis thaliana* (ACX4) and a hydroxybenzoyl CoA thioesterase from *Arthrobacter* spp. [90]. Although this enabled whole-cell microbial production of MAA for the first time, the need to have isobutyrate added to the medium constrains the application of the pathway at an industrial scale. Patents have also been filed describing the production of MMA through transference of a methyl group to methacrylyl-CoA prompted by alcohol acetyl transferases [89,91]. Although several candidate enzymes are put forward in these patents, no evidence has been provided to demonstrate the capability of these enzymes to convert methacrylyl-CoA into an ester of MAA. The sole successful demonstration of MMA production by esterification of methacrylyl-CoA was obtained using whole-protein extracts from *Vitis vinifera*; however, it was not possible to pinpoint which enzyme(s) were performing the observed conversion. The poor annotation of the *V. vinifera* genome sequence and the low conservation among alcohol acetyl transferase-encoding genes also renders it difficult to identify interesting candidates. A notable observation from the retrobiosynthesis work was that, apparently, itaconic acid is not an interesting substrate for the in vivo production of MAA or MMA.

## 7. Conclusions

Microbial production at an industrial scale of various CAs considered of higher interest is already quite advanced, with the production of succinic, lactic or even itaconic acid representing good examples of processes that appear to be on track for a successful industrial implementation. The situation is considerably less advanced for those CAs whose direct porting of the native production pathways to amenable hosts for industrial microbiotechnology is more challenging. For those CAs that are non-natural, the path to microbe-based production is only starting to be constructed; this is also the case for the more interesting industrial derivatives that can be produced from CAs. It is expected that the results reported at an academic scale and the described new-to-nature biosynthetic pathways for various non-natural CAs and derivatives can pave the way for subsequent improvements in yields that could turn production at higher scales feasible and sustainable. The assembly of synthetic pathways to produce non-natural compounds has been greatly accelerated by the use of computer-assisted methods that provide novel combinations of enzymes and substrates and in the specific case of CAs, multiple possibilities have been put forward that require some degree of investment in in vivo assembly. This approach opens the door to the identification of less obvious substrates useful for production, as well as to the identification of more interesting/effective pathways (and this is also true even for molecules that already have implemented pathways). This appears to be the case with methacrylic acid, whose bio-based production from itaconic acid appears highly challenging, despite the obvious chemical similarities. However, this more generalized application of metabolic retrobiosynthesis requires significant efforts to be undertaken, mainly at the enzyme assignment step (still requires a extensive manual mining) to determine which are more interesting candidates to pursue, and necessarily, the selection of which pathways are more interesting to pursue for subsequent tests *in vivo*. The improvement of the genome annotation method and an improvement in the integration of the available information about enzymes into the same tools that perform the metabolic prospection is likely to ease these burdens, increasing the speed at which one can perform successful prospection of metabolic pathways in a relatively straightforward and reliable manner. Despite the bottlenecks, it is clear that the emergence of synthetic biology in industrial microbiotechnology already had and is predicted to continuing impacting microbial production of carboxylic acids thus fostering a reduction in the dependence of fossil fuels.

## Figures and Tables

**Table 1:** CAs identified as more interesting *green* building blocks, according to reports identified by the US DOE [8,10] or by the European research project BREW [9]. Showing the application, the main industrial production method, the already described possibilities of production via microbial fermentation (for those cases in which fermentation is not already the main production method) and the derivatives with the potential to displace oil-derived precursors. Relevant references are provided in the text. MAA-methacrylic acid; MMA-methylmethacrylic acid; THF-Tetrahydrofuran.

CA	Applications	Important Derivatives	Main Industrial Production Method	Microbe-Based Production Alternative
**Malic**	Detergents, food additives, pharmaceuticals, polyesters, solvents	Butanediol, THF, γ-butyrolactone	Hydration of maleic anhydride	Fermentation from renewable resources (Novozymes) using *Aspergillus oryzae*
**Succinic**			Hydrogenation of maleic anhydride, maleic or fumaric	Fermentation at pilot scale using *E. coli* (BioAmber and Myrant)*, S. cerevisiae* (Reverdia*)* and *Basfia succiniciproducens* (Succinity)
**Fumaric**			Synthesis from butane-derived maleic	No industrial process established; production using fermentation with filamentous Fungi reported
**3-Hydroxypropionic**	Superabsorbent, adhesives, surface coatings and paintings	Acrylates and 1,3-propanediol	Hydrolysis of 3-hydroxypropionitrile, hydrolysis of β-propiolactone and oxidation of 1,3- propanediol	No industrial process established; Fermentation from glucose or glycerol reported, mainly using *E. coli* and *Klebsiella* sp.
**Aspartic**	Nutritional supplement in food and animal feed, sweeteners	Polyaspartic, aspartic anhydride, amine butanediol, amine THF, amine butyrolactone	Amination of fumaric acid (enzymatic or with immobilized cells), fermentation with *E. coli* or *Coreynebacterium glutamicum*	-
**Glucaric**	Nylons and polyesters	Lactone, polyglucaric esters and amides	Chemical oxidation with nitric acid	Glucose fermentation using *E. coli* (Kalion)
**Glutamic**	Food additive, potential new polymers	1,5-propanediol, 1,5-propanediacid, 5-amino, 1-butanol	Glucose fermentation using *C. glutamicum*	-
**Itaconic**	Rubber, solvents, acrylates, detergents, superabsorbents, drug delivery polymers, dental materials	MAA, MMA, polyesters, poly-itaconic acid polymers and styrene-butadiene	Glucose fermentation using *Aspergillus terreus*	-
**Levulinic**	Solvents, polymers, acrylates, herbicides, photodynamic therapy	2-methyl-THF, levulinate esters, 1,4-pentanediol, β-acetoacrylate, lactones, δ-aminolevulinic, diphenolic acid	Acid hydrolysis of crystalline sugars or lignocellulosic residues	No industrial process established; reported production from glucose using *E. coli* or an undefined microbial consortia
**Lactic**	Biodegradable fibers in clothing, furniture and biomaterials	Lactate esters, propylene glycol, acrylates, poly-lactic acid	Fermentation of glucose from corn, cassava and sugarcane using *Lactobacillus* sp.	-
**Acetic**	Food additive, solvent, fibers, filters, cellulose plastics and resins (used in paints, adhesives, coatings and textiles)	Vinyl acetate, acetic anhydride, acetate esters, monochloroacetic acid	Methanol carbonylation; liquid-phase oxidation of aliphatic hydrocarbons; fermentation using acetic acid bacterial (mainly in the vinegar industry)	-
**Citric**	Acidulant, preservative, emulsifier, flavoring additive, sequestrant and buffering agent	-	Starch or glucose fermentation using *A. niger*	-
**Gluconic**	Cleaning and construction industries, food additives including prebiotics	Glucono-lactone, sodium gluconate	Oxidation of glucose; Glucose fermentation using *A. niger*	-
**Adipic**	Nylons and polyesters, plasticizers and lubricants	Esters for polymerization (PVC)	Synthesis from benzene	Fermentation of fatty acid rich-feedstocks (Verdezyne) or glucose (BioAmber) using yeasts (at a pilot scale)
**Acrylic**	Various coatings (decorative, industrial, drug tablets, clothes), adhesives, polishes, carpet backing compounds	Methyl acrylate, ethyl acrylate, butyl acrylate and 2-ethylhexyl acrylate, polyacrylates	Oxidation of propene	Fermentation from renewable feedstocks (Arkema); Fermentation of dextrose and sucrose-based feedstocks (OPXBio and Dow) using *E. coli* (at pilot-scale)
**Glycolic**	Tanning and dyeing agent for textiles, packaging materials	Polyglycolate, polyglicoside, butyl-glycolate	Catalysis from CO_2_ and formaldehyde and hydrolysis of chloroacetic acid	No industrial process established; reported production using *E. coli* (natural producer) or the yeasts *S. cerevisiae* and *Kluveromyces lactis* (engineered producers)
**Muconic**	Plastics industry (automotive and packaging applications), synthetic fibers for textiles or industry (mainly nylon) and food acidifying agent	Adipic, terphthalic acid and trimellitic acid, caprolactam	Catalytic oxidation of cyclohexanol or cyclohexanol/cyclohexanone mixtures	No industrial process established; reported production from degradation of benzene-like xenobiotics and from glucose fermentation using Pseudomonas spp. (natural producers) and *E. coli* and *S. cerevisiae* (engineered hosts)

**Table 2:** Examples of enzyme combinations that have been successfully assembled to enable production of glucaric, muconic, adipic, acrylic and levulinic acids in *E. coli* or *S. cerevisiae.* The data shown here is further complemented in Supplementary Table S1, where it shows the comprehensive list of enzyme combinations published thus far in the assembly of synthetic pathways for the production of the above referred CAs. The numbers in brackets assigned to each non-native enzyme correspond to the steps of the synthetic pathway shown in Figure 2 and Figures 4–6. It is also indicated for each enzyme the corresponding EC number.

**Glucaric acid**																		**Titer**	**Ref**
		**Myo-inositol synthase (1)** **5.5.1.4**				**Myo-inositol oxygenase (2)** **1.13.99.1**							**Glucuronic acid dehydrogenase (3); 1.1.1.305**						
***E. coli***		Scaffolded—*S. cerevisiae* Ino1				Scaffolded—*M. musculus* MIOX							Scaffolded—*P. syringae* Udh					2.5 g/L	[15]
***S. cerevisiae***		Endogenous Ino1				Stabilized *Arabidopsis thaliana* MIOX							*P. syringae* Udh					6 g/L	[18]
**Muconic acid**																		**Titer**	**Ref**
**From Dihydroshikimate**	***E. coli***	**Dihydroshikimate Hydratase (1); 4.2.1.118** *Klebsiella pneumoniae* aroZ				**Protocatechuate decarboxylase (2)** **4.1.1.63** *K. pneumoniae* aroY							**Catechol 1.2-dioxygenase (3) 1.13.11.1** *Acinetobacter calcoaceticus* CatA					38.6 g/L ^●^	[29]
	***S. cerevisiae***	*P. anserina* aroZ				*Talaromyces atroroseus* GDC1							*A. radioresistens* CatA					1.24 g/L	[28]
**From chorismate**	***E. coli***	**Isochorismate synthase (8)**; **5.4.4.2** Endogenous EntC			**Isochorismate pyruvate lyase (11); 4.2.99.21** *P. fluorescens* PchB						**Salicylate monoxygenase (12)**; **1.14.13.1** *P. putida* nahG					**Catechol 1.2-dioxygenase (3) 1.13.11.1** *P. putida* CatA		1.5 g/L	[30]
**From anthranilate**	***E. coli***	**Anthranilate 1.2-dioxygenase (14)** **1.14.12.1** *P. aeruginosa* AntABC						**Catechol 1.2-dioxygenase (3)** **1.13.11.1** *P. putid*a CatA										389.96 mg/L	[30]
**From tyrosine**	***E. coli***	**Tyrosine phenol lyase** **(15);4.1.99** *Citrobacter brakii* tutA				**Phenol hydrolyase (7)1.14.13.7** *P. steutzeri* PhKLMOP							**Catechol 1.2-dioxygenase (3) 1.13.11.1** *P. putida* CatA					186 mg/L	[31]
**Adipic acid**																		**Titer**	**Ref**
**Reverse adipate route**	***S. cerevisiae***	**3-Oxoadipyl-CoA thiolase** **(1); 2.3.1.174** *T. fusca* Tfu_0875	**3-Hydroxyadipyl-CoA dehydrogenase (2) 1.1.1.35** *T. fusca* Tfu_2399				**2,3-Dehydroadipyl-CoA hydratase** **(3); 4.2.1.17** *T. fusca* Tfu_0067					**Adipyl-CoA dehydrogenase** **(4);1.1.1.35** *T. fusca* Tfu_1647					**Adipyl-CoA thioesterase (5)** *T. fusca* Tfu_2577 and 2576	3.83 mg/L	[32]
Reverse β-oxidation followed by ω-reduction *	***E. coli***	**3-ketoacyl-CoA thiolase (6)2.3.1.16** *C. necator* BktB	**Trans-enoyl-CoA reductase (7) 1.3.1.44** *E. gracilis* Ter				**ω-Hydroxylase (8) 1.14.15.3** *P. putida* AlkBGT					Alcohol dehydrogenase (9) * *Acinetobacter* spp. ChnD					Aldehyde dehydrogenase (10) * *Acinetobacter* spp. ChnE	-	[33]
**2-oxopimelic route**	***E. coli***	**2-oxoglutaric elongation to 2-oxopimelic (11)** *A. vinelandii* nifV (2.3.3.14) and *M. aeolicus Nankai* AksD (4.2.1.114), AksE (4.2.1.33), and AksF *				**2-Oxopimelic decarboxylase (12)4.1.1.72** *Lactococcus lactis* KdcA							**Adipic semi-aldehyde oxidation (13)** Unknown endogenous enzyme					0.3 g/L	[34]
**From muconic acid**	***S. cerevisiae***	**DHS hydratase** **4.2.1.118** *P. anserina* aroZ		**Protocatechuate decarboxylase; 4.1.1.63** *Enterobacter cloacae* aroY					**Catechol 1,2-dioxygenase1.13.11.1** *C. albicans* HQD2						**Enoate reductase*****(22)*****1.3.1.31** *Bacillus coagulans* MAR (MAR-BC)			2.6 mg/L	[35]
**Acrylic acid**																		**Titer**	**Ref**
**From glycerol**	***E. coli***	**Glycerol-3-P phosphatase3.1.3.21** *S. cerevisiae* Gpp2	**Glycerol** **Dehydratase** **4.2.1.30** *K. pneumoniae* DhaB				**Aldehyde** **Dehydrogenase** **1.2.1.16** *C. necator* GabD4					**CoA transferase** **2.8.3.8** *C. necator* YdiF					**CoA** dehydratase* A. flavithermus Aflv_0566	0.12 g/L	[36]
**Levulinic acid**																		**Titer**	**Ref**
**From 3-oxoadipic**	***E. coli***	**Succinyl-CoA transferase** **2.8.3.18** *C.kluyveri* Cat1				**β-ketoadipyl-CoA thiolase (1)** **2.3.1.174** Endogenous PaaJ				**3-Oxoadipyl-CoA transferase** **(2)**; **2.8.3.6** *P. putida* PcaIJ				**3-Oxoadipic acid decarboxylase** **(3);4.1.1.4** *C. acetobutylicum* Adc				159 mg/L	[37]
		**PCA synthesis (4)** *P. putida* Fcs (6.2.1.34), Ech (4.1.2.61), Vdh (1.2.1.67), VanAb (1.14.13.82), PobA (1.14.13.2)				**Dearomatization pathway (5)** *P. putida* PcaGH (1.13.11.3), PcaB (5.5.1.2), PcaC (4.1.1.44), PcaD (3.1.1.24)							**3-Oxoadipic Acid decarboxylase** **(3); 4.1.1.4** *C. acetobutylicum* Adc					455 mg/L	[38]
**Methacrylic acid**																		**Titer**	**Ref**
**Through Isobutyryl-CoA**	***E. coli***	**Isobutyryl-CoA synthase (1) 6.2.1.3** *P. chlorpraphis* AcsA				**Acyl-CoA oxidase** **(2); 1.3.3.6** *A. thaliana* ACX4							**Hydroxybenzoyl-CoA thioesterase (3); 3.1.2.23** *Arthrobacter* spp. 4HBT					~250 µM	[39]

^●^ The very different yield, compared to the others in synthetic pathways that utilized the same enzymes, results from this study having been undertaken in a bioreactor with fed-batch supplementations of glucose. * Denotes enzymatic assignments that result from promiscuity, and therefore it is not shown as an attributed E.C. number.

## Data Availability

Supplementary Tables S1 and S2 and references provided therein provided support to the information described in the manuscript.

## Supplementary Materials

The following are available online at https://www.mdpi.com/article/10.3390/jof7121020/s1, Table S1: Complete list of enzymatic combinations to assemble new-to-nature pathways leading to the production of the acids reviewed here; Table S2: Overview of most known new-to-nature pathway prospecting tools.
