# Supplementary material for: Implementation of Synthetic Pathways to Foster Microbe-Based Production of Non-Naturally Occurring Carboxylic Acids and Derivatives
Source: J Fungi (Basel). 2021 Nov 29;7(12):1020. doi: 10.3390/jof7121020 (PMC8706239; doi:10.3390/jof7121020)
Supplement: Supplementary file 1 [file jof-07-01020-s001.zip › jof-1417831-supplementary.pdf]

Supplementary Table S1. Assembled synthetic pathways for production of glucaric, adipic, muconic, acrylic and levulinic acids in *E. coli* and/or in *S. cerevisiae* from sugars. The table shows the compiled information about the combinations of enzymes that had been reported to be utilized in synthetic pathways aiming the production of the referred CAs. Note that endogenous enzymes of the host, as well as additional metabolic engineering strategies that could have been used to improve flux through the synthetic pathways (such as the co-expression of adjuvant enzymes) are not described in this table. Each enzymatic step is numbered so that it matches the graphical representations of the pathways in Figs. 2, 4, 5 and 6,. The combination of enzymes suggested by in silico metabolic retrobiosynthesis, albeit not validated in vivo, are highlighted in light grey. For those steps with no specific enzymes assigned, the E.C. number or the E.C. sub(sub)class are indicated. PCA – Protocatechuate;

Glucaric acid					
	**Myo-inositol synthase (1)**	**Myo-inositol oxygenase (2)**	**Glucoronic acid dehydrogenase (3)**	**Titer**	**Ref**
E. coli	*S. cerevisiae* Ino1	*Mus musculus* MIOX	*Pseudomonas syringae* Udh	1.13 g/L	[1]
	Scaffolded - *S. cerevisiae* Ino1	Scaffolded – *M. musculus* MIOX	Scaffolded - *P. syringae* Udh	2.5 g/L	[2]
S. cerevisiae	Endogenous Ino1	*M. musculus* MIOX	*P. syringae* Udh	1.6 g/L	[3]
	Endogenous Ino1	*Arabidopsis thaliana* MIOX	*P. syringae* Udh	0.94 g/L	[3]
	Endogenous Ino1	Stabilized *Arabidopsis thaliana* MIOX	*P. syringae* Udh	6 g/L	[4]
	Endogenous Ino1	*Talaromyces marneffei* MIOX	*P. syringae* Udh	1.76 g/L	[5]
P. pastoris	Endogenous Ino1	*M. musculus* MIOX	*P. putida* Udh	6.61 g/L	[6]

Muconic acid					
From dihydroshikimate					
	**DHS hydratase (1)**	**Protocatechuate decarboxylase (2)**	**Catechol 1,2-dioxygenase (3)**	**Titer**	**Ref**
E. coli	*K. pneumoniae* aroZ	*K. pneumoniae* aroY	*Acinetobacter calcoaceticus* CatA	38.6 g/L	[7]
	Bacillus thurigiensis AroZ	*K. pneumoniae* aroY	*P. putida* CatA	1.59 g/L	[8]
	C. glutamicum QsuB	*K. pneumoniae* aroY	*P. putida* CatA	1.58 g/L	[9]

		Bacillus thurigiensis AroZ	*K. pneumoniae* aroY	*Acinetobacter calcoaceticus* CatA		1.79 mg/L	[10]
		Fused *E. coli* AroD- *B. thurigiensis* AroZ	*K. pneumoniae* aroY	*Acinetobacter calcoaceticus* CatA		1.34 g/L	[8]
		K. pneumoniae aroZ	*K. pneumoniae* aroY	Engieered *Acinetobactor sp.* CatA		1.53 g/L	[11]
	S. cerevisiae	*Podospora anserina* aroZ	*K. pneumoniae* aroY	*Acinetobacter radioresistens* CatA		1.56 mg/L	[12]
		P. anserina aroZ	*K. pneumoniae* aroY	*Candida albicans* HQD2		~20 mg/L	[13]
		P. anserina aroZ	*Enterobacter cloacae* aroY	*C. albicans* HQD2		141 mg/L	[13]
		Neurospora crassa AroZ	*Commensalibacter intestini* AroY	*Cupriavidus necator* CatA		65 mg/L (from xylose)	[14]
		P. anserina aroZ	*Talaromyces atroroseus* GDC1	*A. radioresistens* CatA		1.24 g/L	[15]
	P. putida	*Bacillus cereus* AsbF	*Enterobacter cloacae* aroY	Endogenous CatA		4.92 g/L	[16]
	C. glutamicum	Endogenous qsuB	*K. pneumoniae* aroY	Endogenous CatA		4.5 g/L	[17]
				From chorismate			
from 4-hydroxybenzoic acid	E. coli	**Chorismate pyruvate lyase (4)**	**4-HBA hydrolyase (5)**	**Protocatechuate decarboxylase (2)**	**Catechol 1,2-dioxygenase (3)**		
		Endogenous UbiC	PobA *P. Putida*	*K. pneumoniae* aroY	*P. putida* CatA	170 mg/L	[18]
		Endogenous UbiC	PobA *P. Aeruginosa*	*K. pneumoniae* aroY	*P. putida* CatA	819 mg/L	[9]
		**Chorismate pyruvate lyase (4)** Endogenous UbiC	**4-HBA decarboxylase (6)** *K. pneumoniae* kpdBCD	**Phenol hydrolase (7)** *Pseudomonas steutzeri* PhKLMOP	**Catechol 1,2-dioxygenase (3)** *P. putida* CatA	230 mg/L	[9]
from 2,3-dihydroxy benzoate	*E. coli*	**Isochorismate synthase (8)** Endogenous EntC and EntB	**2,3-dihydro-2,3-DHB dehydrogenase (9)** Endogenous EntA	**2,3-DHB decarboxylase (10)** *P. pneumonia* EntX	**Catechol 1,2-dioxygenase (3)** *P. putida* CatA	480 mg/L	[19]
from salicylate	*E. coli*	**Isochorismate Synthase (8)**	**Isochorismate pyruvate lyase (11)**	**Salicylate monoxygenase (12)**	**Catechol 1,2-dioxygenase (3)**		

		Endogenous EntC	*P. Fluorescens PchB*	*P. putida* nahG	*P. putida* CatA		1.5 g/L	[20]
		Fused *E. coli* AroC- E. coli *MenF*	*P. Aeruginosa* PchB	*P. putida* nahG	*P. putida* CatA		4.45 g/L	[8]
		**Isochorismate synthase (8)** Endogenous EntC	**Isochorismate pyruvate lyase (11)** *P. Aeruginosa* PchB	**Salicylate decarboxylase (13)** *T. Monoliforme* Sdc	**Phenol hydrolase (7)** *Pseudomonas steutzeri* PhKLMOP	**Catechol 1,2-dioxygenase (3)** *P. putida* CatA	484 mg/L	[9]
From anthranilate								
	E. coli	**Anthranilate 1,2-dioxygenase (14)** *P. Aeruginosa* AntABC	**Catechol 1,2-dioxygenase (3)** *P. putida* CatA				389.96 mg/L	[19]
From tyrosine								
	E. coli	**Tyrosine phenol lyase (15)** *Citrobacter brakii* tutA	**Phenol hydrolyase (7)** *Pseudomonas steutzeri* PhKLMOP	**Catechol 1,2-dioxygenase (3)** *P. putida* CatA			186 mg/L	[9]

Adipic acid								
From 3-oxoadipyl-coA								
		**3-Oxoadipyl-CoA thiolase (1)**	**3- Hydroxyadipyl-CoA dehydrogenase (2)**	**2,3-Dehydroadipyl-CoA hydratase (3)**	**Adipyl-CoA dehydrogenase (4)**	**Adipyl-CoA thioesterase (5)**	**Titer**	**Ref**
Reverse adipate route	*E. coli*	Endogenous PaaJ	Endogenous PaaH	Endogenous PaaF	*C. acetobutylicum* Bcd	*M. musculus* Acot8	12 µg/L	[21]
		Endogenous PaaJ	Endogenous PaaH	Endogenous PaaF	*Acinetobacter bayly* DcaA	*A. Baylyi* TesB	6 mg/L	[22]
		Endogenous PaaJ	*C. acetobutylicum* Hbd	*C. acetobutylicum* Crt	*Euglena gracilis* Ter	*C. acetobutylicum* Ptb and Buk1 (phosphate transfer)	31 µg/L	[23]
		T. fusca Tfu_0875	*T. fusca* Tfu_2399	*T. fusca* Tfu_0067	*T. fusca* Tfu_1647	*T. fusca* Tfu_2577 and 2576	0.3 g/L	[24]
		T. fusca Tfu_0875	*T. fusca* Tfu_2399	*T. fusca* Tfu_0067	Engineered *T. fusca* Tfu_1647	*T. fusca* Tfu_2577 and 2576	0.23 g/L	[25]
		Endogenous PaaJ	Endogenous PaaH	Endogenous PaaF	*Treponema denticola* TdTer	*M. musculus* Acot8	170 mg/L	[26]
	S. cerevisiae	*T. fusca* Tfu_0875	*T. fusca* Tfu_2399	*T. fusca* Tfu_0067	*T. fusca* Tfu_1647	*T. fusca* Tfu_2577 and 2576	3.83 mg/L	[27]

	-	*3-Oxoadipyl-CoA Thiolase* 2.3.1.174 (validated)	3-oxoadipyl-CoA transferase (6) 2.8.3.6 (validated)	3-oxoadipate dehydrogenase (7) 1.1.1.172 (validated)	3-Hydroxyadipate Dehydratase (8) ?	2-Enoate reductase (9) ?	-	[28]
From PCA	*E. coli*	**PCA synthesis**. *P. putida* Fcs, Ech, Vdh, VanAb, PobA	**De-aromatization pathway** *P. putida* PcaGH, PcaB, PcaC,PcaD	**β-ketoadipic acid succinyl-CoA transferase** *P. putida* PcaIJ	**3-Oxo group reduction** *R. eutropha* PaaH1 and Ech and *T. denticola* TdTer	**CoA removal** *C. acetobutylicum* Ptb1 and Buk1	9.5 mg/L (from lignin)	[29]
Reverse β-oxidation followed by ω-reduction								
	E. coli	***3-ketoacyl-CoA thiolase (6*** *)Ralstonia eutropha* BktB	**Trans-enoyl-CoA Reductase (7)** *E. gracilis* Ter	**ω-Hydroxylase (8)** *P. putida* AlkBGT	**Alcohol Dehydrogenase (9)** *Acinetobacter spp* ChnD	**Aldehyde Dehydrogenase (10)** *Acinetobacter spp* ChnE	-	[30]
From 2-oxoadipic acid								
Through 2-Oxopimelic	*E. coli*	**2-oxoglutaric elongation (11)** AksA, AksD, AksE and AksF (*A. Vinelandii, & M. aeolicus Nankai and V. fluvialis*)		**2-Oxopimelic Decarboxylase (12)** *Lactococcus lactis* KdcA	**Adipic semialdehyde oxidation (13)** Unknown endogenous enzyme		0.3 g/L	[31]
Through 2-hydroxyadipic	-	**2-Hydroxyadipate dehydrogenase (14)** 1.1.1.-	**2-Hydroxyadipyl-CoA synthase (15)** 6.2.1.-	**2-Hydroxyadipyl-CoA (16)** 4.2.1.-	***Adipyl-CoA dehydrogenase (4)*** 1.3.1.44 (validated above)	***Adipyl-CoA thioesterase (5)*** 6.2.1.- (validated above)	-	[28]
From lysine	-	**Lysine transaminase (17)** 2.6.1.36 (validated)	**Allysine Oxidase (18)** 1.2.1.31 (validated)	**2-aminoadipic transaminase (29)** ?	**2-hexenedioic reductase (20)** E. coli NemA and S. pastorianus Oye1 (not validated)		-	[32]
From muconic acid								
	S. cerevisiae	**DHS Hydratase** *P. anserina* aroZ	**Protocatechuate Decarboxylase** *Enterobacter cloacae*	**Catechol 1,2-dioxygenase** *C. albicans* HQD2	**Enoate reductase** *Bacillus coagulans* MAR (MAR-BC)		2.6 mg/L	[33]

		aroY					
E. coli	***Isochorismate Synthase*** Endogenous EntC	***Isochorismate Pyruvate Lyase*** *P. Fluorescens PchB*	***Salicylate Monoxygenase*** *P. putida* nahG	***Catechol 1,2-dioxygenase*** *P. putida* CatA	***Enoate reductase*** *C. acetobutylicum ER*	12.4 mg/L	[34]
From oxalyl-CoA							
-	**Methyltransferase** 2.1.1.197	**Condensation** 2.3.1.16/180	**Reductase, Dehydratase and Reductase** 1.1.1.100/36, 4.2.1.119 and 1.3.8/10/38/44	**Pymeloyl-ACP Methyl Esterase** 3.1.1.85		-	[35]
From acetyl-CoA (Polyketide route)							
-	**Polyketide synthase** *Streptomyces spp.* PKS Bor (validated in vitro)						[36]
Acrylic Acid							
from glycerol							
E. coli	**Glycerol-3-P phosphatase** *S. cerevisiae* Gpp2	**Glycerol dehydratase** *K. pneumoniae* DhaB	**Aldehyde dehydrogenase** *C. necator* GabD4	**CoA transferase** *C. necator* YdiF	**CoA dehydratase** *A. flavithermus* Aflv_0566	0.12 g/L	[37]
from 3-hydroxypropionate/4-hydroxybutyrate							
E. coli	**Malonyl-CoA reductase** *Metallosphaera sedula* Mcr	**Malonate semialdehyde Reductase** *M. sedula* Msr	**3-Hydroxypropionyl-CoA synthetase** *M. sedula* 3Hpcs	**3-Hydroypropionyl-CoA dehydratase** *M. sedula* 3Hpcd		13.28 mg/L	[38]
from β-alanine							
E. coli	**Aspartate -1-Decarboxylase** *C. glutamicum* PanD	**β-alanine CoA transferase (Act)** *Clostridium propionicum* Act	**β-alanyl- CoA:ammonia lyase** *C. propionicum* Acl2	**Propionate CoA Transferase** *C. necator* YciA		237 mg/L	[39]
Levulinic Acid							
from 3-Oxoadipic acid							
	**Succinyl-CoA transferase**	**β-ketoadipyl-CoA thiolase (1)**	**3-Oxoadipyl-CoA transferase (2)**	**3-Oxoadipic acid decarboxylase (3)**			
E. coli	*Clostridium kluyveri* Cat1	Endogenous PaaJ	*P. putida* PcaIJ	Unknown endogenous		48 mg/L	[26]

					enzyme			
		C.kluyveri Cat1	Endogenous PaaJ	*P. putida* PcaIJ	*S. habrochaites* Mks1		71 mg/L	[26]
		C.kluyveri Cat1	Endogenous PaaJ	*P. putida* PcaIJ	*C. acetobutylicum* Adc		159 mg/L	[26]
		**PCA synthesis (4)**<br>*P. putida* Fcs, Ech, Vdh, VanAb, PobA	**Dearomatization pathway (5)**<br>*P. putida* PcaGH, PcaB, PcaC,PcaD	**3-Oxoadipic Acid decarboxylase (3)**<br>*C. acetobutylicum* Adc			455 mg/L	[29]
	-	**3-DHS Dehydratase (6)**<br>4.2.1.118 (validated)	**PCA Decarboxylase, Catechol 1,2-Dioxygenase, Muconate cycloisomerase, muconolactone δ-isomerase and 3-oxoadipate enol-lactonase**<br>4.1.1.6, 1.3.11.1, 5.5.1.1., 5.3.3.4 and 3.1.1.24 (validated)			**3-Oxoadipic acid decarboxylase (3)**<br>4.1.1.112 (validated above)		[40]
from pyruvic acid and acetaldehyde								
	-	**Aldolase (7)**<br>4.1.3.17 (validated)	**4-hydroxy-2-oxopentanoic acid oxidase (8)**<br>?	**2,4-Dioxovaleric dehydratase (8)**<br>?	**4-Oxo-2-Pentenoic reductase (8)**<br>?	**NADPH dehydrogenase (8)**<br>1.6.99.1 (validated)		[41]
from D-ALA								
	-	**D-ALA transaminase (9)**<br>2.6.1.62/105		**Diaminovaleric ammonia lyase (10)**<br>4.3.1.15				[40]
	-	**Glutamate semialdehyde aminomutase (11)** 5.4.3.8 (requires engineering)		**Diaminovaleric ammonia lyase (10)**<br>4.3.1.15				[40]
from lysine degradation								
		**5-Amino valeric aminomutase (12)**<br>5.4.3.3/5		**4-Aminovaleric transaminase (13)**<br>2.6.1.18				[40]
**Methacrylic Acid**								
from methacrylyl-CoA								
Through Isobutyryl-CoA	*E. coli*	**Isobutyryl-CoA synthase (1)**<br>*P. chlorpraphis* AcsA	**Acyl-CoA oxidase (2)**<br>*A. Thaliana* ACX4	**Hydroxybenzoyl-CoA thioesterase (3)**<br>*Arthrobacter spp.* 4HBT			~ 250 μM	[42]
Through	-	**4-Hydroxyisobutyryl-CoA**	**3-Hydroxyisobutyryl-**	**Methacrylyl-CoA**				[43]

3-Hydroxyisobutyryl-CoA		**mutase (4)** 5.4.99.-	**CoA dehydratase (5)** 4.2.1.55/17	**transferase (3)** 6.2.1.13 (validated above)			
Through 2-Hydroxyisobutyryl-CoA	-	**Acetoacetyl-CoA thiolase (6)** 2.3.1.9 (validated)	**Acetoacetyl-CoA reductase (7)** 1.1.1.35 (validated)	**3-Hydroxyisobutyrate mutase (8)** 5.4.99.-	**2-Hydroxyisobutyryl-CoA dehydratase (9)** 4.2.1.54/167/157	**Methacrylyl-CoA transferase (3)** 6.2.1.13 (validated above)	[43]
from mesaconic acid							
	-	**Mesaconate Decarboxylase (10)** 4.1.1.6/77					
from 3-hydroxyisobutyric acid							
	-	**4-Hydroxyisobutyryl-CoA mutase (11)** 5.4.99.-		**3-Hydroxyisobutyryl-CoA hydrolase (12)** 3.1.2.4 (validated)		**3-Hydroxyisobutyrate dehydratase (13)** 1.1.1.291	[43]
	-	**Methylmalonyl-CoA reductase (14)** 1.2.1.-		**3-Hydroxyisobutyrate dehydrogenase (15)** 1.1.1.31 (validated)		**3-Hydroxyisobutyrate dehydratase (13)** 1.1.1.291	[43]
from 3-aminoIsobutyric acid							
	-	**Methylmalonyl-CoA Reductase (16)** 1.2.1.-		**3-Aminoisobutyrate Transaminase (17)** 2.6.1.22 (validated)		**3-Aminoisobutyrate ammonia lyase (18)** 4.3.1.1.	[43]

Supplementary Table S2: Described computational tools for assisted in silico pathway prospection. The table described the reaction rules used in the retrobiosynthesis search used by the tools, as well as the criteria used to rank the obtained candidate pathways. Information on the enzyme assignment performed is also indicated as well as the accompanying reference and the date of release of the tool.

Metabolic retrobiosynthesis tool	Reaction rules	Criteria used to rank pathways	Level of enzyme assignment	Comments	Ref	Year
**UM-BBD**	From literature on functional group transformations	Not performed by the tool	Identifies a specific enzyme	Applied to xenobiotic degradation; only allows KEGG compounds	[44]	2004
**BNICE**	From KEGG, BEM representation at the 3rd EC level	Not performed by the tool	Identifies a reaction rule to which the enzyme belongs to	-	[45]	2005
**Biopath finder**	Generated from all known classes of enzymes that operate on carbohyydrates	Pathway length	Identifies a reaction rule to which the enzyme belongs to	Only applied to central carbon metabolism	[46]	2010
**PathPred**	RDM patterns from KEGG RPAIR	Similarity of synthetic substrates with native ones	Identifies a KEGG Reaction	Applied to xenobiotic degradation, only allows KEGG compounds	[47]	2010
**-**	From KEGG	Binding site covalence, chemical similarity between synthetic and native substrates, thermodynamics, pathway length, and organism specificity	Identifies the full EC number	-	[48]	2010
**Simpheny**	From general E.C. information	Yield, pathway length, number of non-native steps, number of novel/synthetic steps and thermodynamics	Not described	Integration with GEMs	[49]	2011
**GEM-Path**	From general E.C. information. SMILES/SMARTS	Theoretical productivity	Identifies the full EC number	Integration with GEMs	[50]	2014
**XTMS**	Molecular signatures	Enzyme efficiency, toxicity, yield and thermodynamics	Based on the identification of similar reactions	Search tool is Retropath	[51]	2014

**SimIndex and SimZyme**	Chemical fingerprints and BNICE rules	Similarity between the synthetic and the native substrates	Identifies a full EC number	Algorithmic improvement by Byers -Waterman search	[52]	2015
**M-Path**	Reaction feature vectors based on KEGG RPAIR	Similarity between the synthetic and the native substrates	Based on the identification of similar KEGG reactions	Only allows KEGG compounds (in the demo server)	[53]	2015
**ReactPred**	From Metacyc SMILEs/SMARTS	Pathway length, thermodynamics, molecular size and substructure	Identifies a reaction rule	Reaction rules may be generated automatically from other libraries	[54]	2016
**RetroPath 2.0**	From Metacyc, SMILES/SMARTS	Can be done using an external tool (RP2paths)	Identifies a full EC number	-	[55]	2018
**RING**	From KEGG (small subset)	Pathway length	Identifies a reaction rule	Created small manual networks	[56]	2018
**RePrime**	From metRxn, based on molecular signatures and metabolite moieties	Implicit constrains in the MILP optimization problem	Identifies an enzyme ID	Integration w/ GEMs	[57]	2018
**RetSynth**	RetroRules	The min of number of genes added is the MILP objective	Identifies a full EC number	Integration w/ GEMs	[58]	2019
**Transfor-MinER**	From KEGG based on reaction center (RC) and the molecular environment (RCME)	Similarity between the synthetic and the native substrates	Based on similar KEGG reactions	-	[59]	2019
**Retropath RL**	From RetroRules database, accepts user reaction rules	It is not performed directly in the tool but using a separate procedure	Identifies a full EC number	-	[60]	2020

References

1. Moon, T.S.; Yoon, S.-H.; Lanza, A.M.; Roy-Mayhew, J.D.; Prather, K.L.J. Production of glucaric acid from a synthetic pathway in recombinant Escherichia coli. *Applied and environmental microbiology* **2009**, *75*, 589-595, doi:10.1128/AEM.00973-08.
2. Moon, T.S.; Dueber, J.E.; Shiue, E.; Prather, K.L.J. Use of modular, synthetic scaffolds for improved production of glucaric acid in engineered E. coli. *Metabolic Engineering* **2010**, *12*, 298-305, doi:10.1016/j.ymben.2010.01.003.
3. Gupta, A.; Hicks, M.A.; Manchester, S.P.; Prather, K.L.J. Porting the synthetic D-glucaric acid pathway from Escherichia coli to Saccharomyces cerevisiae. *Biotechnology Journal* **2016**, 1201-1208, doi:10.1002/biot.201500563.
4. Chen, N.; Wang, J.; Zhao, Y.; Deng, Y. Metabolic engineering of Saccharomyces cerevisiae for efficient production of glucaric acid at high titer. *Microbial Cell Factories* **2018**, *17*, 67, doi:10.1186/s12934-018-0914-y.
5. Marques, W.L.; Anderson, L.A.; Sandoval, L.; Hicks, M.A.; Prather, K.L.J. Sequence-based bioprospecting of myo-inositol oxygenase (Miox) reveals new homologues that increase glucaric acid production in Saccharomyces cerevisiae. *Enzyme and Microbial Technology* **2020**, 109623, doi:10.1016/j.enzmictec.2020.109623.
6. Liu, Y.; Gong, X.; Wang, C.; Du, G.; Chen, J.; Kang, Z. Production of glucaric acid from myo-inositol in engineered Pichia pastoris. *Enzyme and Microbial Technology* **2016**, *91*, 8-16, doi:https://doi.org/10.1016/j.enzmictec.2016.05.009.
7. Niu, W.; Draths, K.M.; Frost, J.W. Benzene-Free Synthesis of Adipic Acid. *Biotechnology Progress* **2002**, *18*, 201-211, doi:10.1021/bp010179x.
8. Fujiwara, R.; Noda, S.; Tanaka, T.; Kondo, A. Muconic Acid Production Using Gene-Level Fusion Proteins in Escherichia coli. *ACS Synthetic Biology* **2018**, *7*, 2698-2705, doi:10.1021/acssynbio.8b00380.
9. Thompson, B.; Pugh, S.; Machas, M.; Nielsen, D.R. Muconic Acid Production via Alternative Pathways and a Synthetic "metabolic Funnel". *ACS Synthetic Biology* **2018**, *7*, 565-575, doi:10.1021/acssynbio.7b00331.
10. Choi, S.; Lee, H.-N.; Park, E.; Lee, S.-J.; Kim, E.-S. Recent Advances in Microbial Production of cis,cis-Muconic Acid. *Biomolecules* **2020**, doi:10.3390/biom10091238.
11. Han, L.; Liu, P.; Sun, J.; Wu, Y.; Zhang, Y.; Chen, W.; Lin, J.; Wang, Q.; Ma, Y. Engineering catechol 1, 2-dioxygenase by design for improving the performance of the cis, cis-muconic acid synthetic pathway in Escherichia coli. *Scientific Reports* **2015**, *5*, 13435, doi:10.1038/srep13435.
12. Weber, C.; Brückner, C.; Weinreb, S.; Lehr, C.; Essl, C.; Boles, E. Biosynthesis of cis,cis-muconic acid and its aromatic precursors, catechol and protocatechuic acid, from renewable feedstocks by saccharomyces cerevisiae. *Applied and Environmental Microbiology* **2012**, *78*, 8421-8430, doi:10.1128/AEM.01983-12.
13. Curran, K.A.; Leavitt, J.M.; Karim, A.S.; Alper, H.S. Metabolic engineering of muconic acid production in Saccharomyces cerevisiae. *Metabolic Engineering* **2013**, *15*, 55-66, doi:10.1016/j.ymben.2012.10.003.
14. Liu, T.; Peng, B.; Huang, S.; Geng, A. Recombinant xylose-fermenting yeast construction for the co-production of ethanol and cis,cis-muconic acid from lignocellulosic biomass. *Bioresource Technology Reports* **2020**, *9*, 100395, doi:https://doi.org/10.1016/j.biteb.2020.100395.
15. Brückner, C.; Oreb, M.; Kunze, G.; Boles, E.; Tripp, J. An expanded enzyme toolbox for production of cis, cis-muconic acid and other shikimate pathway derivatives in Saccharomyces cerevisiae. *FEMS Yeast Research* **2018**, *18*, doi:10.1093/femsyr/foy017.
16. Johnson, C.W.; Salvachúa, D.; Khanna, P.; Smith, H.; Peterson, D.J.; Beckham, G.T. Enhancing muconic acid production from glucose and lignin-derived aromatic compounds via increased protocatechuate decarboxylase activity. *Metabolic Engineering Communications* **2016**, *3*, 111-119, doi:https://doi.org/10.1016/j.meteno.2016.04.002.

17. Shin, W.S.; Lee, D.; Lee, S.J.; Chun, G.T.; Choi, S.S.; Kim, E.S.; Kim, S. Characterization of a non-phosphotransferase system for cis,cis-muconic acid production in Corynebacterium glutamicum. *Biochemical and Biophysical Research Communications* **2018**, *499*, 279-284, doi:10.1016/j.bbrc.2018.03.146.
18. Sengupta, S.; Jonnalagadda, S.; Goonewardena, L.; Juturu, V. Metabolic engineering of a novel muconic acid biosynthesis pathway via 4-hydroxybenzoic acid in Escherichia coli. *Applied and Environmental Microbiology* **2015**, *81*, 8037-8043, doi:10.1128/AEM.01386-15.
19. Sun, X.; Lin, Y.; Yuan, Q.; Yan, Y. Biological Production of Muconic Acid via a Prokaryotic 2,3-Dihydroxybenzoic Acid Decarboxylase. *ChemSusChem* **2014**, *7*, 2478-2481, doi:10.1002/cssc.201402092.
20. Lin, Y.; Sun, X.; Yuan, Q.; Yan, Y. Extending shikimate pathway for the production of muconic acid and its precursor salicylic acid in Escherichia coli. *Metabolic Engineering* **2014**, *23*, 62-69, doi:https://doi.org/10.1016/j.ymben.2014.02.009.
21. Babu, T.; Yun, E.J.; Kim, S.; Kim, D.H.; Liu, K.H.; Kim, S.R.; Kim, K.H. Engineering Escherichia coli for the production of adipic acid through the reversed β-oxidation pathway. *Process Biochemistry* **2015**, *50*, 2066-2071, doi:10.1016/j.procbio.2015.09.018.
22. Kallscheuer, N.; Gätgens, J.; Lübcke, M.; Pietruszka, J.; Bott, M.; Polen, T. Improved production of adipate with Escherichia coli by reversal of β-oxidation. *Applied Microbiology and Biotechnology* **2017**, *101*, 2371-2382, doi:10.1007/s00253-016-8033-3.
23. Yu, J.-L.; Xia, X.-X.; Zhong, J.-J.; Qian, Z.-G. Direct biosynthesis of adipic acid from a synthetic pathway in recombinant <i>Escherichia coli</i>. *Biotechnology and Bioengineering* **2014**, *111*, 2580-2586, doi:10.1002/bit.25293.
24. Zhao, M.; Huang, D.; Zhang, X.; Koffas, M.A.G.; Zhou, J.; Deng, Y. Metabolic engineering of Escherichia coli for producing adipic acid through the reverse adipate-degradation pathway. *Metabolic Engineering* **2018**, *47*, 254-262, doi:10.1016/j.ymben.2018.04.002.
25. Yang, J.; Wei, Y.; Li, G.; Zhou, S.; Deng, Y. Computer-aided engineering of adipyl-CoA synthetase for enhancing adipic acid synthesis. *Biotechnology Letters* **2020**, *42*, 2693-2701, doi:10.1007/s10529-020-02978-y.
26. Cheong, S.; Clomburg, J.M.; Gonzalez, R. Energy- and carbon-efficient synthesis of functionalized small molecules in bacteria using non-decarboxylative Claisen condensation reactions. *Nature Biotechnology* **2016**, *34*, 556-561, doi:10.1038/nbt.3505.
27. Zhang, X.; Liu, Y.; Wang, J.; Zhao, Y.; Deng, Y. Biosynthesis of adipic acid in metabolically engineered Saccharomyces cerevisiae. *Journal of Microbiology* **2020**, *58*, 1065-1075, doi:10.1007/s12275-020-0261-7.
28. Burgard, A.P.; Pharkya, P.; Osterhout, R.E. Microorganisms for the production of adipic acid and other compounds. **2012**.
29. Oh, M.-K.; Kim, B.S.; Chang, M.W.; Lo, T.-M.; Hwang, I.Y.; Cho, H.-S.; Fedora, R.E.; Chng, S.H.; Choi, W.J. Biosynthesis of Commodity Chemicals From Oil Palm Empty Fruit Bunch Lignin. *Frontiers in Microbiology* **2021**, doi:10.3389/fmicb.2021.663642.
30. Clomburg, J.M.; Blankschien, M.D.; Vick, J.E.; Chou, A.; Kim, S.; Gonzalez, R. Integrated engineering of β -oxidation reversal and ω -oxidation pathways for the synthesis of medium chain ω -functionalized carboxylic acids. *Metabolic Engineering* **2015**, *28*, 202-212, doi:10.1016/j.ymben.2015.01.007.
31. Turk, S.C.H.J.; Kloosterman, W.P.; Ninaber, D.K.; Kolen, K.P.A.M.; Knutova, J.; Suir, E.; Schürmann, M.; Raemakers-Franken, P.C.; Müller, M.; De Wildeman, S.M.A.; et al. Metabolic Engineering toward Sustainable Production of Nylon-6. *ACS Synthetic Biology* **2016**, *5*, 65-73, doi:10.1021/acssynbio.5b00129.
32. Karlsson, E.; Shin, J.H.; Westman, G.; Eriksson, L.A.; Olsson, L.; Mapelli, V. In silico and in vitro studies of the reduction of unsaturated α , β bonds of trans -2-hexenedioic acid and 6-amino- trans -2-hexenoic acid – Important steps towards biobased production of adipic acid. *plos* **2018**, 1-23.
33. Raj, K.; Partow, S.; Correia, K.; Khusnutdinova, A.N.; Yakunin, A.F.; Mahadevan, R. Biocatalytic production of adipic acid from glucose using engineered Saccharomyces cerevisiae. *Metabolic Engineering Communications* **2018**, *6*, 28, doi:10.1016/J.METENO.2018.02.001.
34. Sun, J.; Raza, M.; Sun, X.; Yuan, Q. Biosynthesis of adipic acid via microaerobic hydrogenation of cis,cis-muconic acid by oxygen-sensitive enoate reductase. *Journal of Biotechnology* **2018**, *280*, 49-54, doi:10.1016/j.jbiotec.2018.06.304.

35. Botes, A.L.; Van Eck, A.C. Methods of producing 6-carbon chemicals via methyl-ester shielded carbon chain elongation. **2013**.
36. Hagen, A.; Poust, S.; Rond, T.D.; Fortman, L.; Katz, L.; Petzold, C.J.; Keasling, J.D. Engineering a Polyketide Synthase for In Vitro Production of Adipic Acid. **2016**, doi:10.1021/acssynbio.5b00153.
37. Chu, H.S.; Ahn, J.-h.; Yun, J.; Choi, I.S.; Nam, T.-w.; Myung, K. Direct fermentation route for the production of acrylic acid. *Metabolic Engineering* **2015**, *32*, 23-29, doi:10.1016/j.ymben.2015.08.005.
38. Liu, Z.; Liu, T. Production of acrylic acid and propionic acid by constructing a portion of the 3-hydroxypropionate/4-hydroxybutyrate cycle from Metallosphaera sedula in Escherichia coli. *Journal of Industrial Microbiology & Biotechnology* **2016**, *43*, 1659-1670, doi:10.1007/s10295-016-1843-6.
39. Ko, Y.S.; Kim, J.W.; Chae, T.U.; Song, C.W.; Lee, S.Y. A novel biosynthetic pathway for the production of acrylic acid through β-alanine route in escherichia coli. *ACS Synthetic Biology* **2020**, *9*, 1150-1159, doi:10.1021/acssynbio.0c00019.
40. Vila-Santa, A.; Islam, M.A.; Ferreira, F.C.; Prather, K.L.J.; Mira, N.P. Prospecting Biochemical Pathways to Implement Microbe-Based Production of the New-to-Nature Platform Chemical Levulinic Acid. *ACS Synthetic Biology* **2021**, *10*, 724-736, doi:10.1021/acssynbio.0c00518.
41. Zanghellini, A.L. Fermentation route for the production of levulinic acid, levulinate esters and valerolactone and derivatives thereof. 2019.
42. Eastham, D.W.J.; Ronald, G.; Poliakoff, M.; Huddle, T.A. A process for the production of methacrylic acid and its derivatives and polymers produced therefrom. 2011.
43. Burgard, A.P.; Burk, M.J.; Osterhout, R.E.; Pharkya, P. Microorganisms for the production of methacrylic acid. 2008.
44. Bo, K.H.; Ellis, L.B.M.; Wackett, L.P. Encoding microbial metabolic logic: Predicting biodegradation. *J. Ind. Microbiol. Biotechnol.* **2004**, *31*, 261-272, doi:10.1007/s10295-004-0144-7.
45. Hatzimanikatis, V.; Li, C.; Ionita, J.A.; Henry, C.S.; Jankowski, M.D.; Broadbelt, L.J. Exploring the diversity of complex metabolic networks. *Bioinformatics* **2005**, *21*, 1603-1609, doi:10.1093/bioinformatics/bti213.
46. Noor, E.; Eden, E.; Milo, R.; Alon, U. Central Carbon Metabolism as a Minimal Biochemical Walk between Precursors for Biomass and Energy. *Mol. Cell* **2010**, *39*, 809-820, doi:10.1016/j.molcel.2010.08.031.
47. Moriya, Y.; Shigemizu, D.; Hattori, M.; Tokimatsu, T.; Kotera, M.; Goto, S.; Kanehisa, M. PathPred: an enzyme-catalyzed metabolic pathway prediction server. *Nucleic Acids Res.* **2010**, *38*, W138-143, doi:10.1093/nar/gkq318.
48. Cho, A.; Yun, H.; Park, J.H.; Lee, S.Y.; Park, S. Prediction of novel synthetic pathways for the production of desired chemicals. *BMC Systems Biology* **2010**, *4*, doi:10.1186/1752-0509-4-35.
49. Yim, H.; Haselbeck, R.; Niu, W.; Pujol-Baxley, C.; Burgard, A.; Boldt, J.; Khandurina, J.; Trawick, J.D.; Osterhout, R.E.; Stephen, R.; et al. Metabolic engineering of Escherichia coli for direct production of 1,4-butanediol. *Nat. Chem. Biol.* **2011**, *7*, 445-452, doi:10.1038/nchembio.580.
50. Campodonico, M.A.; Andrews, B.A.; Asenjo, J.A.; Palsson, B.O.; Feist, A.M. Generation of an atlas for commodity chemical production in Escherichia coli and a novel pathway prediction algorithm, GEM-Path. *Metab. Eng.* **2014**, *25*, 140-158, doi:10.1016/j.ymben.2014.07.009.
51. Delépine, B.; Carbonell, P.; Faulon, J.L. XTMS in action: Retrosynthetic design in the extended metabolic space of heterologous pathways for high-value compounds. *Lecture Notes in Computer Science (including subseries Lecture Notes in Artificial Intelligence and Lecture Notes in Bioinformatics)* **2014**, *8859*, 256-259, doi:10.1007/978-3-319-12982-2_21.
52. Pertusi, D.A.; Stine, A.E.; Broadbelt, L.J.; Tyo, K.E.J. Efficient searching and annotation of metabolic networks using chemical similarity. *Bioinformatics* **2015**, *31*, 1016-1024, doi:10.1093/bioinformatics/btu760.
53. Araki, M.; Cox, R.S.; Makiguchi, H.; Ogawa, T.; Taniguchi, T.; Miyaoku, K.; Nakatsui, M.; Hara, K.Y.; Kondo, A. M-path: A compass for navigating potential metabolic pathways. *Bioinformatics* **2015**, *31*, 905-911, doi:10.1093/bioinformatics/btu750.

54. Sivakumar, T.V.; Giri, V.; Park, J.H.; Kim, T.Y.; Bhaduri, A. ReactPRED: a tool to predict and analyze biochemical reactions. *Bioinformatics* **2016**, *32*, 3522-3524, doi:10.1093/bioinformatics/btw491.
55. Delépine, B.; Duigou, T.; Carbonell, P.; Faulon, J.L. RetroPath2.0: A retrosynthesis workflow for metabolic engineers. *Metab. Eng.* **2018**, *45*, 158-170, doi:10.1016/j.ymben.2017.12.002.
56. Gupta, U.; Le, T.; Hu, W.S.; Bhan, A.; Daoutidis, P. Automated network generation and analysis of biochemical reaction pathways using RING. *Metab. Eng.* **2018**, *49*, 84-93, doi:10.1016/j.ymben.2018.07.009.
57. Kumar, A.; Wang, L.; Ng, C.Y.; Maranas, C.D. Pathway design using de novo steps through uncharted biochemical spaces. *Nat. Commun.* **2018**, *9*, doi:10.1038/s41467-017-02362-x.
58. Whitmore, L.S.; Nguyen, B.; Pinar, A.; George, A.; Hudson, C.M. RetSynth: Determining all optimal and sub-optimal synthetic pathways that facilitate synthesis of target compounds in chassis organisms. *BMC Bioinformatics* **2019**, *20*, 1-14, doi:10.1186/s12859-019-3025-9.
59. Tyzack, J.D.; Ribeiro, A.J.M.; Borkakoti, N.; Thornton, J.M. Exploring Chemical Biosynthetic Design Space with Transform-MinER. *ACS Synth. Biol.* **2019**, *8*, 2494-2506, doi:10.1021/acssynbio.9b00105.
60. Koch, M.; Duigou, T.; Faulon, J.L. Reinforcement Learning for Bioretrosynthesis. *ACS Synth. Biol.* **2020**, doi:10.1021/acssynbio.9b00447.
